# Recent Progress in Nucleic Acid Aptamer-Based Biosensors and Bioassays

**DOI:** 10.3390/s8117050

**Published:** 2008-11-07

**Authors:** Wendy Mok, Yingfu Li

**Affiliations:** 1 Department of Biochemistry and Biomedical Sciences, McMaster University, 1200 Main Street West, Hamilton, Ontario, L8N 3Z5, Canada; E-mail: mokwk@mcmaster.ca; 2 Department of Chemistry, McMaster University, 1280 Main Street West, Hamilton, Ontario, L8S 4M1, Canada

**Keywords:** Aptamers, biosensors, bioassays

## Abstract

As the key constituents of the genetic code, the importance of nucleic acids to life has long been appreciated. Despite being composed of only four structurally similar nucleotides, single-stranded nucleic acids, as in single-stranded DNAs and RNAs, can fold into distinct three-dimensional shapes due to specific intramolecular interactions and carry out functions beyond serving as templates for protein synthesis. These functional nucleic acids (FNAs) can catalyze chemical reactions, regulate gene expression, and recognize target molecules. Aptamers, whose name is derived from the Latin word *aptus* meaning “to fit”, are oligonucleotides that can bind their target ligands with high affinity and specificity. Since aptamers exist in nature but can also be artificially isolated from pools of random nucleic acids through a process called *in vitro* selection, they can potentially bind a diverse array of compounds. In this review, we will discuss the research that is being done to develop aptamers against various biomolecules, the progress in engineering biosensors by coupling aptamers to signal transducers, and the prospect of employing these sensors for a range of chemical and biological applications. Advances in aptamer technology emphasizes that nucleic acids are not only the fundamental molecules of life, they can also serve as research tools to enhance our understanding of life. The possibility of using aptamer-based tools in drug discovery and the identification of infectious agents can ultimately augment our quality of life.

## 1. Introduction

As the carriers of genetic information and templates for guiding protein synthesis, nucleic acids have long been renowned for being the fundamental molecules of life. The discovery of nucleic acid sequences with catalytic and regulatory activities over the past decades illuminated a whole new array of functions that can be conducted by these simple, yet multifaceted macromolecules. These “molecular workhorses”, appropriately coined “functional nucleic acids” (FNAs), encompass a range of DNA and RNA sequences that can act as enzymes, recognition elements, and molecular switches. The current collection of FNAs consists of a plethora of naturally occurring RNA sequences that participate in regulating gene expression and controlling cellular processes. Their presence provides a mean for cells to respond promptly to physical and chemical signals in order to maintain homeostasis. Advances in nucleic acid synthesis and modification introduce artificial functional sequences, which include single-stranded DNAs and sequences with non-natural nucleotides, into the growing pool of FNAs. In conjunction with mining for novel FNAs, current research efforts have been diverted to finding applications for these sequences, such as converting existing FNAs into *in vitro* and *in vivo* tools. Since its debut less than a decade ago, research in the conversion of aptamers, one class of these FNAs, into biosensors is quickly maturing into a league of their own.

Aptamers and aptamer-based biosensors will be the focus of this review. We will begin with a discussion on the origins, properties, and diversity in the ensemble of aptamer biosensors currently available to us. These sensors are attainable by combining aptamers against different targets with various signal-transducing platforms. Afterward, we will explore the prospects of integrating these sensors into existing biochemical techniques and applying them in assays to quantify biologically relevant compounds and macromolecules, detect toxins and infectious agents, and identify potential drug compounds. This list of possible applications goes to show that these biosensors hold great promise for research, diagnostics, and treatment.

## 2. The properties of nucleic acids

**Figure 1.**
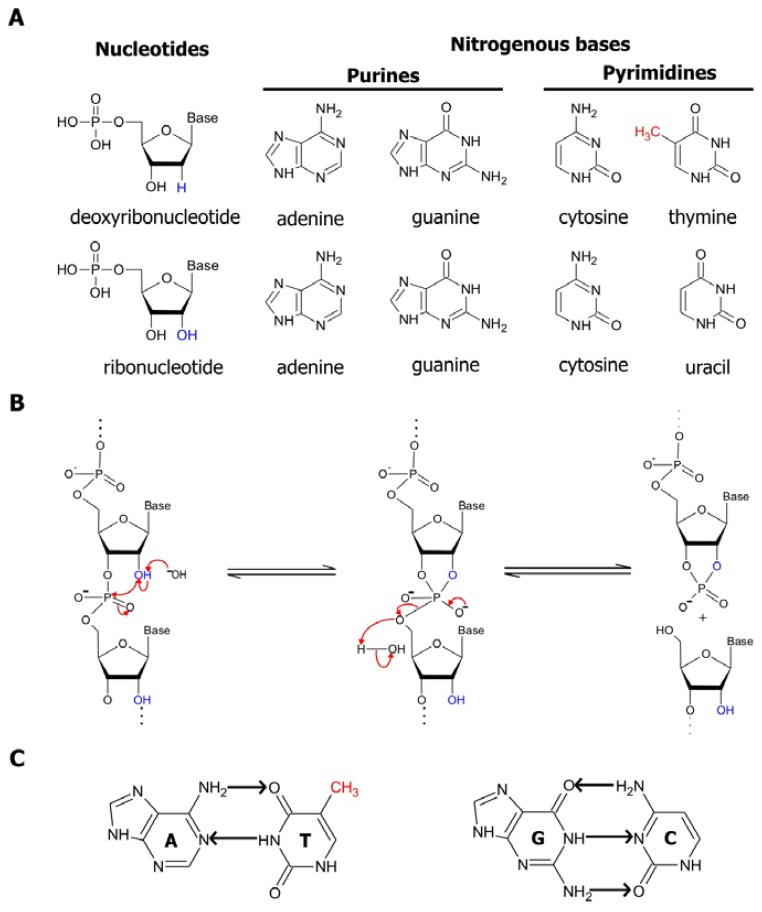
Properties of nucleic acids. a) The structures of deoxyribonucleotides and ribonucleotides, the building blocks of DNA and RNA, are illustrated. b) Pathway of base-mediated degradation of RNA. c) Hydrogen bonding between purine and pyrimidine nitrogenous bases.

In nucleic acids, four basic building blocks known as nucleotides, each comprised of a common sugar phosphate and one of four distinct nitrogenous bases, are joined together in different combinations and permutations by phosphodiester linkages to create an assortment of polymers with unique sequences. The structural properties of these polymers have been comprehensively examined in numerous texts and publications, one of which is a book by Bloomfield, Crothers, and Tinoco, Jr. [1]. Hence, we will only present a brief discussion of these properties here. DNA, which is predominantly recognized for being the reservoir for genetic information, is made up of deoxyribonucleotide subunits. Each of these subunits contains a deoxyribose (a pentose sugar ring) attached to either an adenine, cytosine, guanine or thymine nitrogenous base (Figure 1a, top panel). As it was disclosed in the classic manuscript by Watson and Crick in 1953, two strands of DNA are coiled together in an antiparallel manner, forming the double helical structures that reside in cells [2]. On the other hand, RNA, the molecular cousin of DNA, is single-stranded. Moreover, in RNA the thymine base is replaced by uracil, and the ribonucleotide subunits making up RNA is assembled with a ribose sugar, which differs from deoxyribose by having a hydroxyl (OH) group in its 2 position in place of hydrogen (Figure 1a, bottom panel).

The sugars present in deoxyribonucleotides and ribonucleotides contribute to the differences in stability between DNA and RNA polymers. The presence of the 2′ hydroxyl group in riboses promotes the internal phosphoester transfer in RNA, thereby enhancing the susceptibility of RNA to cleavage and degradation. This transesterification reaction is accelerated by the presence of divalent magnesium ions, high temperature or hydroxide ions, which increase the rate of RNA cleavage by approximately 100,000-fold. Basic conditions favor the deprotonation of the 2′-OH and the nucleophilic attack by the 2′ oxygen on the adjacent phosphorous center via an S_N_2 mechanism, resulting in a cleavage product with a 2′,3′-cyclic phosphate terminus and another product with a 5′-hydroxyl terminus (Figure 1b) [3]. Inside the cell, the stability of RNA is further deterred by enzymes that are involved in the degradation of nucleic acids. Known as nucleases, these enzymes function by breaking the phosphate ester backbones of the polymers. In eukaryotic cells, the majority of these enzymes are responsible for the degradation of RNAs. These RNA-targeting enzymes ensure the viability of cells by modulating messenger RNA (mRNA) turnover, controlling gene expression, obliterating RNAs from viral pathogens, and removing aberrant mRNAs [1]. The existence of these destabilizing factors attributed to the intrinsic design of RNAs and cellular mechanisms emphasizes the need to incorporate modifications to protect the integrity of RNAs when engineering RNA-based molecular tools.

The self-assembly of RNA sequences into complex structures serves as a method of protecting the RNA from RNases and from spontaneous cleavage by preventing the in-line attack of the 2′-hydroxyl group on the adjacent phosphorous center. The structures formed within and between single-stranded nucleic acid polymers is mainly a result of the interactions between their nitrogenous bases. These bases are decorated with a number of complementary hydrogen bond acceptors and donors. The common mechanism of association between two complementary sequences is by Watson-Crick base-pairing, in which purine nitrogenous bases (guanine and adenine) form hydrogen bonds with the pyrimidine nitrogenous bases (cytosine, thymine, and uracil) at their Watson-Crick interfaces (Figure 1c). Since guanines interact with cytosines through 3 hydrogen bonds, while adenines only form 2 hydrogen bonds with thymines or uridines, G:C base pairings are more stable than A:T or A:U interactions. In addition to the Watson-Crick interactions, base-pairing between nucleotides can occur through other mechanisms, such as Hoogsteen base-pairing, which involves the formation of hydrogen bonds between atoms at the Hoogsteen interface of the nitrogenous bases rather than at the Watson-Crick interface [1]. Since the nitrogenous bases are non-polar structures, they may also associate by stacking on each other through hydrogen bonding, hydrophobic interactions, electrostatic forces and van der Waals forces in order to reduce the area exposed to polar solvents. Moreover, the bases can also interact by a combination of hydrogen bonding and base stacking to produce more complex structures. For example, it has been previously demonstrated that within guanine-rich regions of a nucleic acid sequence, four guanine residues can assemble into a square co-planar array known as a guanine quartet through 8 hydrogen bonds. The guanines of these quartets can further be stacked, thus resulting in the formation of a stable quadruplex structure consisting of 4 strands [4]. The various mechanisms of interaction between nitrogenous bases allow for the association of two complementary strands of nucleic acids, as observed in double-stranded DNA helices. These interactions are also the principle instigators of the activity of functional nucleic acids. Not only are they the driving force behind the folding of single-stranded sequences into specific catalytic structures, they grant these sequences the ability to selectively bind their target substrates and ligands, as we will see in subsequent sections.

## 3. Overview of functional nucleic acids

Over the past two and a half decades, many classes of natural and synthetic FNAs have been discovered. Despite being less stable and receiving less attention than DNA, RNAs have been demonstrated to be the more versatile counterpart of the two naturally-occurring nucleic acids. In addition to serving as templates for protein synthesis (as in mRNAs), adapters for transferring the correct amino acids onto growing polypeptide chains (as in transfer RNAs), and components of the cellular organelles (such as ribosomal RNAs), naturally-occurring RNA sequences with regulatory functions have been unveiled. Small RNAs (sRNAs) found in prokaryotes and micro RNAs (miRNAs) found in eukaryotes have been shown to interact with proteins in order to regulate the activities of the proteins or elicit their functions as a part of a protein-RNA complex [5]. Alternatively, they can bind target mRNAs and regulate gene expression by disrupting the stability of the mRNA or altering their translation into proteins [6]. Collectively, these RNA sequences have been found to participate in the regulation of a number of key cellular processes, including metabolism, embryonic growth and development, bacterial stress response and virulence, as well as cellular differentiation and apoptosis [6, 7].

A closer examination inside the cell further reveals RNA sequences with catalytic activities. These RNA enzymes, called ribozymes, can catalyze reactions at rates that are comparable to those of their protein counterparts. Through a process known as *in vitro* selection, which we will describe in detail later on in the manuscript, artificial ribozymes have been isolated, thus broadening the array of reactions that can be catalyzed by these RNA enzymes. Such reactions range from simple intramolecular phosphodiester transfers, such as site-specific self-cleavage and self-splicing, to more sophisticated intermolecular reactions, such as the conversion of nucleotides to nucleosides, amide bond synthesis, and the formation of carbon-carbon bonds [8].

The discovery of ribozymes *in vivo* and *in vitro* sparked interest in finding their DNA counterparts. The search for these catalytic DNA sequences in biological systems proved futile, as these sequences are not known to exist in nature. However, using *in vitro* selection, catalytically active DNA sequences may be generated. In contrast to the DNA duplexes that are commonly found in genomes, these catalytic sequences, referred to as deoxyribozymes or DNAzymes, are single-stranded. This allows them to adopt intricate secondary and tertiary structures. Initial selection efforts by Breaker and Joyce back in 1994 led to the first reported DNA enzyme- a metal ion-dependent RNA-cleaving deoxyribozyme [9]. Since then, a number of RNA-cleaving DNA enzymes have been isolated. Besides RNA cleavage, deoxyribozymes that are capable of catalyzing other reactions, such as RNA ligation [10], auto-phosphorylation [11], and porphyrin metallation [12], have been developed [12, 13]. Over the years, selection endeavours have led to the discovery of DNA enzymes that are active under acidic conditions and in the presence of toxic metal ions, demonstrating that these resilient enzymes can retain their stability and functionality under harsh environments [14].

Due to their ability to fold into distinct three-dimensional structures, single-stranded nucleic acids can form receptors and bind target molecules with high specificity and affinity. In reference to the Latin word *aptus* meaning “to fit”, these nucleic acid receptors have been named “aptamers”. Comparable to synthetic ribozymes and deoxyribozymes, synthetic aptamers are generated through *in vitro* selection. This process was behind the production of a repertoire of DNA and RNA aptamers that can interact with a diverse assortment of ligands ranging from ions, small molecules, peptides, single proteins, organelles, viruses, and even whole cells [15]. Their function may also extend beyond target recognition, as revealed by aptamer-based drugs and tools. For example, it has been shown that some aptamers can act as potential antiviral agents by inhibiting proteins that are critical for viral replication or host cell-epitope recognition upon binding. This includes the hemagglutinin protein in the H5N1 strain of influenza [16].

Apart from being synthesized *in vitro*, RNA aptamers have also been identified *in vivo*, as a part of regulatory sequences known as riboswitches. Located in the untranslated regions of mRNAs, riboswitches are comprised of two main domains: an aptamer domain and an expression platform domain. Upon sensing and binding their target metabolites via their aptamer domains, a change in conformation is induced in the expression platform of the riboswitch. Adopting an alternative secondary structure in the sequence may disrupt the stability of the mRNA or affect its transcription, translation, or splicing, which in turn contributes to a change in the expression of the gene downstream of the riboswitch [17, 18]. The regulatory mechanisms of riboswitches illustrate that aptamers can work in conjunction with catalytic sequences or intrinsic secondary structures to control gene expression. To date, close to 20 classes of naturally-occurring riboswitches, each with a different ligand specificity, have been uncovered. Advances in aptamer selection and nucleic acid rational design also enables for the engineering of synthetic riboswitches that are responsive to ligands that are not detected by their natural counterparts [19].

## 4. Selecting for aptamers- borrowing a page from Mother Nature's cook book

The overview of functional nucleic acids presented in the previous section is by no means exhaustive, but it clearly exemplifies the utility of *in vitro* selection in generating nucleic acids with desired properties and functions. This technique was first conceptualized by Sol Spiegleman and his colleagues in the mid-1960s when they were working on the isolation of RNA sequences that are specifically and efficiently replicated by a viral RNA polymerase [20]. By exploiting the substrate specificity, replication machinery, and error-prone nature of the polymerase, the Spiegleman team simulated the three main events of Darwinian evolution-selection, amplification, and mutation-entirely outside the cell as a chemical process [20]. Spiegleman's work was done before molecular cloning came of age, in an era which Sidney Brenner coined B.C. (“Before Cloning”), thus the amplification of target sequences following selection presented a major road block for carrying out *in vitro* selection [20, 21]. This prompted Spiegleman to disregard the applicability and versatility of his method beyond the context of viral replication [20].

The emergence of a new technique known as polymerase chain reaction (PCR) in the 1980s, which allows for the amplification of just about any sequence, opened the flood gate barricading the inundation of functional nucleic acids identified through *in vitro* selection. In 1990, we saw the evolution of the selection process when the labs of Jack Szostak and Larry Gold separately revitalized this technique and applied it in the selection of RNA sequences that can bind a chosen target, leading to the discovery of RNA aptamers [21]. Gold later coined the term SELEX (Selective Evolution of Ligands by Exponential Enrichment) to describe this more sophisticated selection technique. Before the events of natural selection can be mimicked in order to isolate aptamer sequences, a sample of sequences must first be created. The sample for selection is established by the synthesis of a library of random or partially-random sequences. We can take advantage of the intrinsic error rate of low fidelity polymerases to introduce mutations into sequences, and we can enhance sequence diversity by extending these sequences in the presence of unbalanced concentrations of each nucleotide triphosphate (as in error-prone PCR) or under extreme conditions (as in hypermutagenic PCR). However, these methods are insufficient in maintaining a large sequence space [20]. The common approach for generating a library of DNAs in current selection experiments relies on automated synthesis, which involves the use of solutions containing each of the four nucleoside phosphoramidites and traces of each of the other three nucleotides. Employing this technique, we can control the variation at each position of the randomized section of the sequence. To facilitate the use of PCR to amplify the desired sequences, the randomized portion may be flanked by constant sequences, referred to as the primer binding arms, on both sides.

**Figure 2.**
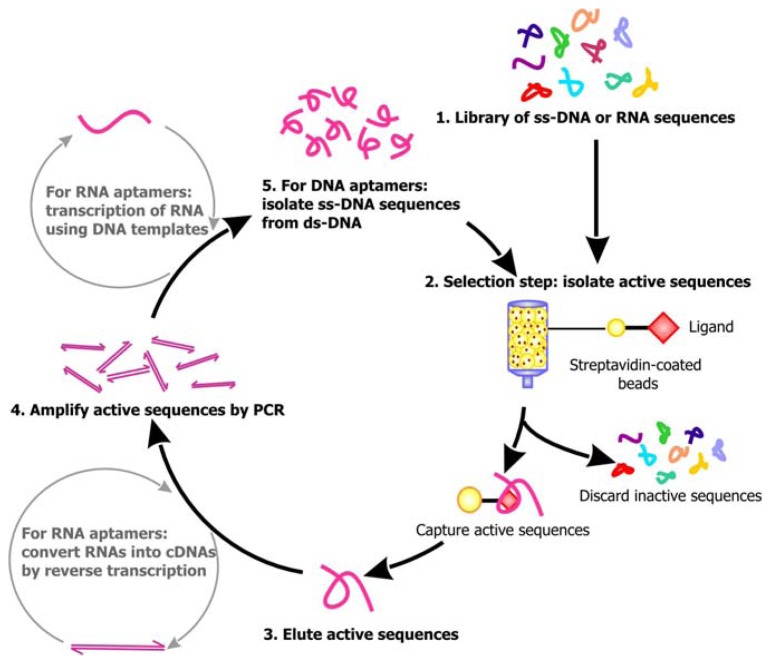
Isolating aptamers by Selective Evolution of Ligands by Exponential Enrichment (SELEX) [22]. 1) Synthesis of a library of sequences for selection. 2, 3) Sequences that can bind a target of interest may be isolated from a library of sequences by affinity chromatography (as illustrated here) or by other selection methods, such as capillary electrophoresis, filtration and immunoprecipitation. 4) The active sequences may be amplified by PCR and 5) subjected to subsequent rounds of selection in order to enrich for the more active and robust sequences. For RNA aptamers, the sequences must be converted into complementary DNAs (cDNAs) prior to PCR amplification. Following PCR, *in vitro* transcription will have to be carried out in order to generate the RNA sequences before another round of selection can occur.

Following library synthesis, the sequences are subjected to selection. A general scheme of the selection process for aptamers is outlined in Figure 2. For the isolation of RNA aptamers, RNA sequences are transcribed from the library of DNAs by *in vitro* transcription, which requires the use of bacterial or viral DNA-dependent RNA polymerase to produce RNA transcripts in the presence of DNA templates and NTPs [22]. Several approaches can then be undertaken to select for the aptamers. Using affinity chromatography, the library of single-stranded DNA sequences or RNA transcripts can be passed through a column comprised of a solid support, such as Streptavidin coated beads, decorated with the target ligand. Active sequences that can bind the ligands will be immobilized on the column as the inactive, unbound sequences are washed away. The active sequences are then eluted from the column. Alternatively, these ligand-bound active sequences may be distinguished from the unbound sequences by capillary electrophoresis (CE), filtration using nitrocellulose filters or immunoprecipitation. In order to enrich for the population of active sequences, PCR may be applied to amplify the eluted sequences. The primers for PCR can anneal to the constant regions flanking the randomized sequences, allowing the target sequences to be copied. The enrichment of RNA aptamers is more complicated, because the RNAs must be first converted into their complementary DNAs (cDNAs) by reverse transcriptase, an RNA-dependent DNA polymerase, before PCR can be conducted. After amplification by PCR, *in vitro* transcription will have to be carried out to regenerate the RNA aptamers. Since the enzymes used for PCR may be error-prone, mutations may be introduced into the sequences during amplification. Following PCR, the candidate aptamer sequences are subjected to a subsequent round of affinity capture and amplification. Typically, to obtain an active sequence of 40-50 nt, 4 to 10 rounds of selection will have to be carried out, because even if a large number of sequences in the library are active, they only represent a small proportion of the initial sequence pool. Thus, several rounds of selection is required before the background noise is minimized and the robust active sequences that are stable enough to survive through the selection process dominate the final pool of sequence.

Although selection promotes the “survival of the fittest”, the aptamer sequences that are generated by *in vitro* selection are rarely the fittest of all the possible sequences. The main restriction to finding the “perfect” sequence lies in the limited area of sequence space that can be sampled in each selection experiment. For practicality reasons, the library size of a standard selection experiment encompasses approximately 10^14^ to 10^16^ molecules. While this may appear to be a vast number of sequences, this value only permits the randomization of approximately 27 positions in a sequence. In other words, when the length of a sequence exceeds 27 nt, it becomes impractical to cover all the variations at every position of the sequence [23]. Due to these limitations, it is common to isolate an active, ligand-bound sequence from the initial library and resynthesize a new library based on the selected (parent) sequence. One method of producing the second library is by partially randomizing each base of the active sequence. For this approach, solutions containing a high percentage of one nucleoside phosphoramidite, in which the base of the nucleoside coincides with the nucleotide at the specific position of the parent sequence, is doped with the other three nucleotides, and this mixture is then used for synthesis. Another technique that can be applied to randomize the sequence is hypermutagenic PCR, as mentioned previously. After creating this new library, the selection process can be repeated, allowing the aptamer sequence to be optimized.

Over the years, new editions of the existing scheme have surfaced, providing investigators with more versatile selection strategies. The counter selection approach, which involves the removal of sequences that may non-specifically interact with the solid supports used for affinity capture or bind to an undesired target prior to selection, can prohibit the isolation of off-target aptamers [24]. Moreover, it has enabled for the generation of aptamers that can distinguish an abnormal or cancerous cell type from the wild-type cell type [25]. Using the parallel selection method, investigators can input a complex target, such as cell fragments, membranes or whole cell preparation, as the SELEX target in place of purified soluble forms of individual proteins [24]. The end product of this single selection experiment would be a collection of aptamers that can bind multiple targets. The aptamers resulting from one selection experiment can be re-subjected to a new round of selection against a different target or a different isoform of the previous target. This process, known as sequential selection, has been reported to produce aptamers with dual-specificity. In other words, they may recognize multiple forms of the same target [24].

*In vitro* selection efforts in the P.C. (“Post-Cloning”) era has contributed to the discovery of a variety of nucleic acid enzymes, whether they are ribozymes or deoxyribozymes, as well as a toolbox of over 150 high-affinity aptamers. Today, nearly 18 years after the birth of the first generation of aptamers, this method is still extensively used and the family of these ligand-binding nucleic acid sequences is still rapidly expanding.

## 5. Putting aptamers to work

Owing to their high affinity and specificity, the function of aptamers has been compared to that of protein antibodies. Like antibodies, the use of aptamers as detection tools is becoming increasingly popular in research. The ease of aptamer synthesis and the increased stability of nucleic acids compared to proteins render it especially advantageous to develop aptamers for specific targets in lieu of raising antibodies against the same targets [26]. Since aptamers can be generated as a chemical process through *in vitro* selection, allowing for the bypass of biological systems in their synthesis, aptamers against non-immunogenic agents or compounds that may be toxic to cells can be developed. Moreover, the binding site of aptamers is determined by the investigator, whereas the epitope bound by antibodies is dictated by the immune system of the organism in which the antibody is raised. This grants the investigator the freedom to choose the binding sites on protein targets based on their knowledge of the protein's interactions and activities when designing aptamers. It also ensures that the activity of aptamers synthesized in different batches would remain consistent. The stability of these structured nucleic acid sequences offers them a long shelf life and tolerance to the harsh, non-physiological conditions that may be necessary for *in vitro* diagnostic assays. Unlike proteins, nucleic acids can refold back into their initial conformation after the dissociation of their ligand and are not sensitive to permanent denaturation following high temperature insults, thus making them reusable. To make the use of aptamers even more enticing, they do not induce an immune response when used *in vivo*, and antidote sequences that can reverse the inhibitory effects of an aptamer can readily be developed.

**Figure 3.**
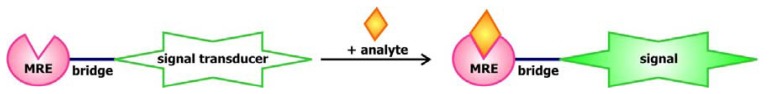
Anatomy of biosensors.

The use of oligonucleotides for the detection of complementary nucleic acid sequences is hardly a novel invention. Back in 1975, E.M. Southern published a technique, commonly referred to as Southern blotting, which combines the use of gel electrophoresis to separate DNA sequence fragments and oligonucleotide probes to identify target fragments [27]. Later, we saw spin-offs of Southern blotting in techniques such as Northern blotting, which allows for the detection of target RNA fragments, reverse transcription-PCR (RT-PCR), in-situ hybridization, and microarray assays. Thanks to advances in the field of aptamer research, we can now use nucleic acids to detect much more than complementary sequences. Modifications to existing aptamers can be made in order to convert these “molecular pacmen” into analytical devices. Biosensors, which are used to detect biologically-relevant molecules, represent one type of these devices [28]. As illustrated in Figure 3, the anatomy of biosensors is quite simplistic and can generally be subdivided into the molecular recognition element (MRE) and the signal transducer. Using the MRE, the sensor detects and binds a biological molecule of interest. The change in binding state of the MRE is subsequently communicated either directly or through a “bridge” element to the signal transducer, prompting the transducer to produce a signal to report the interaction between the sensor and the target. Based on this blueprint, aptamer-based biosensors can be engineered by linking aptamers selected against a target of interest to signal-producing entities, which can emanate optical, acoustic, mechanical, or electric signals. As emphasized previously, aptamers share striking functional similarities with protein receptors and antibodies, and they are superior to proteins in terms of stability and ease of synthesis. As such, aptamer biosensors have the potential to replace the use of proteins as reporters for detecting and quantifying specific analytes in conventional biological assays (or bioassays).

## 6. Optical sensors- where seeing is believing

Although it has been a decade since the realization of the first fluorescence-based aptamer biosensor [29], fluorophores remain one of the most prevalent signal transducers to be incorporated into aptamer biosensors, primarily thanks to the facile conjugation of fluorophores onto nucleic acid sequences and the convenience of detecting the fluorescence signals. Since then, different prototypes of fluorescence signaling aptamer sensors have been developed. The design and characteristics of these sensors are extensively discussed in a previous review by Nutiu and Li [30]. Here, a brief synopsis of the assortment of fluorescence signaling aptamer biosensors that are currently available is presented in table 1.

Despite the triviality of linking fluorophores and quenchers onto existing aptamers in order to convert them into sensors, it has been advised that profound care should be undertaken to minimize disruptions to the aptamer sequences when adding these moieties, because even minor modifications to their structures could obliterate their affinity and specificity. Often, the aptamer sequences have to be altered or optimized following the addition of these signaling elements to restore their functions [40]. Moreover, it has been argued that since these fluorescence signaling sensors are labeled nucleic acids, which cannot be expressed endogenously, they are impractical for intracellular applications [41]. To overcome these short-comings, several groups have worked to improve the existing fluorescence signaling approach and to create novel optical signaling domains.

**Figure 4.**
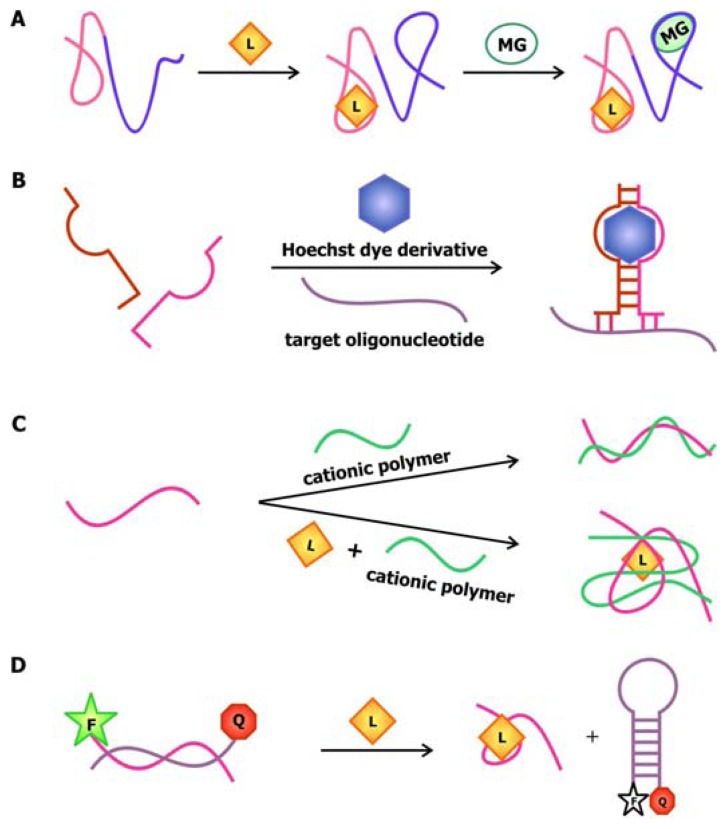
Designs of other optical biosensors. a) The modular aptamer sensor in which the analyte sensing aptamer is linked to a malachite green (MG) binding aptamer, which serves as the signal transducer [41]. b) The Hoechst dye derivative-binding aptamer [42]. c) The aptamer-polymer complex system [44]. d) The trans-acting molecular beacon (in purple) [40].

In 2004, Stojanovic and Kolpashchikov developed modular aptamer sensors in which the MRE and the signal transducer are both comprised of aptamers (Figure 4a), thus eliminating the need to modify the aptamer with fluorophores and quenchers [41]. In this allosteric system, aptamers against an analyte of interest, whether it is ATP, flavin mononucleotide (FMN) or theophylline, are linked to an aptamer specific for malachite green. The binding of the analyte of interest to the MRE would subsequently induce the binding of malachite green to its corresponding aptamer. The quantum yield of the bound malachite green, which is often used as a fluorescent dye, increases up to 2000-fold compared to its unbound state. Therefore, the signal generated by malachite green upon binding serves as a reporter for the presence of the target.

In addition to using malachite green as a reporter in this chimeric aptamer sensor, other fluorescent dyes, especially those that specifically intercalate into double-stranded nucleic acids, have been used to report structural reorganization of the aptamer following ligand binding. Recently, Sando and colleagues reported the use of an aptamer against a modified DNA binding dye as a sensor for target nucleic acid sequences [42]. Prior to selecting for the aptamer, modifications were made to the conventional DNA-staining Hoechst dye, so that the dye would no longer bind its ds-DNA targets. The team then proceeded to select for aptamers that would specifically bind this Hoechst dye derivative. This aptamer was subsequently divided into a two-stranded entity with sequences that are complementary to the target oligonucleotide on each side (Figure 4b). Thus, the aptamer would only be assembled and interact with the Hoechst dye derivative in the presence of the target sequence, resulting in an increase in fluorescence.

The anionic nature of nucleic acid backbone enables the hybridization of cationic polymers to the sequences. This property may be exploited to generate aptamer biosensors with a *trans*-acting signal transducer, in which the transducer is not conjugated onto the aptamer. Making use of the fluorimetric or colorimetric properties of water-soluble cationic polymers such as polythiophene and poly[9,9′-bis-(6′-(*N*,*N*,*N*-trimethylammonium)hexyl)fluorene-*co-alt*-4,7-(2,1,3-benzothiadiazole) dibromide] (PFBT), the signal originating from this sensor depends on the association and dissociation of the aptamer-polymer complex. In the sensor developed by Ho and Leclerc (Figure 4c), the free, unfolded conformation of the thrombin aptamer is accessible to polythiophene [43]. However, thrombin binding disrupts the electrostatic interactions between the aptamer and the polymer, while favoring the association between the polymer and the aptamer-target complex. Since the polymer binds to the complex through a different conformation, a change in the optical signal produced by the polymer is detected. The use of polymers as signal transducers has recently been applied to the microarray technology [44]. In this design, uncharged peptide nucleic acid (PNA) probes, in which the monomers are comprised of a nitrogenous bases linked to a peptide backbone, are immobilized onto a solid phase. Since these probes are neutral, they cannot associate with the positively-charged PFBT polymer until they are bound by their complementary, negatively-charged nucleic acid sequence. This polymer-based detection approach allows for sensing of relevant nucleic acid sequences using-label free probes.

An alternative method to introducing a *trans*-acting signal transducer is presented in a novel aptamer biosensor engineered by Li and Ho. In this construct, the attachment of the signal transducer relies on the hybridization between the aptamer sequence and its antisense competitor sequence [40]. The design of this antisense sequence mimics that of the molecular beacon in the sense that its ends are modified with a fluorophore and a quencher. As illustrated in Figure 4d, in the absence of the target, the antisense sequence hybridizes to the aptamer. This separates the fluorophore and the quencher, resulting in a detectable fluorescence signal. The binding of the ligand to the aptamer displaces the competitor sequence, allowing it to adopt its “closed” configuration, thus promoting fluorescence quenching. This approach and the aforementioned polymer-based signaling approach exemplifies the utility and effectiveness of *trans*-acting signal transducers in communicating target detection by the MRE while bypassing the need to modify the aptamers.

## 7. Immobilized optical sensors- all for one signal

In the optical sensors described previously, the aptamers act as individuals, where each one is independently associated with a signal transducer in a one-to-one ratio. In the immobilized sensors presented here, on the other hand, the aptamers are team players with multiple entities being conjugated onto a single signal transducer. Ligand binding alters the surface properties of the transducer, generating a signal in the form of a change in color, fluorescence intensity or characteristics of waves emanating from these surfaces.

**Figure 5.**
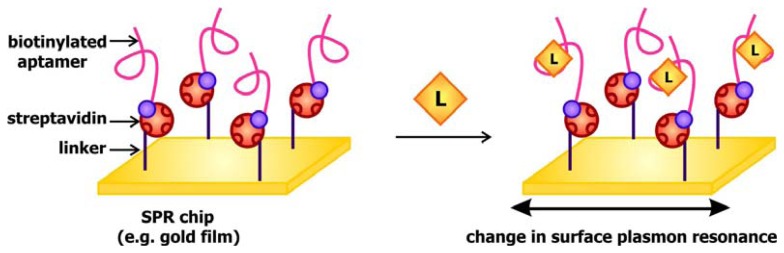
Design of aptamer-based biosensors that signal by surface plasmon resonance (SPR) [45, 46]. Ligand binding to this sensor may be detected by monitoring the change in SPR.

When light strikes the surface of a metal, especially silver and gold, at a particular angle, it can excite electrons on the surface, causing them to resonate and be propagated along the metal. This phenomenon, known as surface plasmon resonance (SPR), may be used to generate a signal to communicate target detection. The signal transducer used in SPR-based aptamer biosensors is generally gold covered disks coated with streptavidin. The presence of streptavidin, a biotin binding protein, enables the immobilization of biotinylated aptamers (Figure 5). When these chips are exposed to their targets, the interaction between the aptamers and their ligands would alter the local refractive index, and in turn change the surface plasmon resonance in response to excitation by the incident light. This fluctuation may be detected by monitoring the change in SPR angle, which is defined as the angle at which the reflected light reaches a minimum intensity, following ligand binding. This system has been previously applied by Tombelli and colleagues for the detection of the HIV-1 Tat (trans-activator of transcription) protein [45] and by Tang and colleagues, who developed a sensor decorated with anti-thrombin DNA aptamers [46]. These groups further demonstrated that the sensor chips may be rinsed with 2M NaCl solutions to prompt ligand dissociation following target detection, rendering them reusable.

The detection of changes in SPR and surface plasmon coupling does not always depend on sophisticated instruments as in the aforementioned sensors. In the case of gold nanoparticles (AuNPs), changes in SPR associated with the transition from the colloidal dispersion state to the aggregation state contribute to a red to violet color change that is observable with the naked eye [47]. This unique property of AuNPs was recognized by mankind as early as the Middle Ages, when these particles were used in tainting stain glass and embellishing decorations with vibrant red and blue colors although the exact mechanism behind the color change was a mystery [48]. In modern times, these particles are actively studied by the scientific community and new applications for them are constantly emerging.

**Figure 6.**
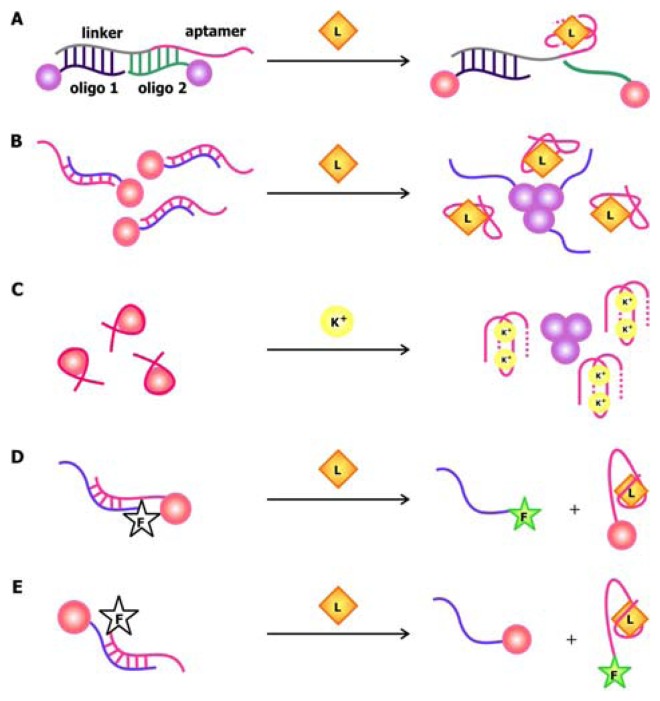
Gold nanoparticles (AuNP)-based biosensors. When these particles are dispersed in solution, a red color is observed. However, when the AuNPs aggregate, the color changes the violet. a) AuNP signaling by crosslinking method [50]. b) AuNP signaling by non-crosslinking method [51]. c) Signaling using unmodified AuNPs [52]. d,e) Signaling by using AuNPs as fluorescence quenchers [53]. The fluorophore can either be modified on the complementary oligonucleotide (d) or on the aptamer (e).

The development of oligonucleotide-modified AuNPs as probes for the selective and sensitive detection of complementary sequences by Mirkin and his coworkers back in 1997 painted the possibility of chemically conjugating these particles with nucleic acids and exploiting their unique distance-dependent optical properties for signaling [49]. Making use of oligonucleotide hybridization and AuNPs, Liu and Lu designed ATP and cocaine aptamer biosensors that use AuNPs as signal transducers [50]. In this system, the aptamers are connected to a linker sequence. The nanoparticles are either functionalized with a sequence that is complementary to the 5′ end of the linker (oligo 1) or one that is complementary to the 3′ end of the linker and part of the aptamer (oligo 2), as shown in Figure 6a. When the target is absent, the linker and aptamer bind to both oligo 1 and oligo 2, thereby crosslinking the AuNPs and forming aggregates that appear violet when suspended in solution. To accommodate their targets, the aptamers adopt folded structures, which displace oligo 2 while the linkers are still bound to oligo 1. This leads to the breakdown of the aggregates and a violet-to-red color change in solution.

Recently, Zhao and colleagues developed an aptamer biosensor that relies on salt-induced, noncrosslinking AuNP aggregation [51]. The AuNPs used in this sensor are functionalized with oligonucleotides that are complementary to part of the aptamer (Figure 6b). The aptamers are bound to their complementary counterparts in the absence of their cognate ligand, but quickly dissociate from them when their targets are present. Since unhybridized AuNPs are less stable at a specific salt concentration compared to their hybridized counterparts, they aggregate and result in a red to violet color change in solution. Even though the salt-induced aggregation phenomenon of AuNPs is also the basis of signal transduction in the potassium biosensor by the Fan group, their sensor is unique in the sense that it involves the use of unmodified particles [52]. This is possible due to the selective interaction of AuNPs with free K^+^ aptamers that adopt a random-coil conformation but not with bound aptamers that fold into a structured G-quartet (Figure 6c). The association of the nanoparticles with the single-stranded aptamers stabilizes them in solution with high salt and prevents their aggregation.

The versatility of AuNPs is epitomized in the “signal on” thrombin sensors by Wang *et al.* in which AuNPs are used as fluorescence quenchers (Figure 6d,e) [53]. Together, these examples demonstrate that gold nanoparticles may be used as signal transducers in association with a wide range of aptamers in order to produce sensitive and selective biosensors with easily detectable signals.

The incorporation of quantum dots (QD), another type of nanoarchitecture, into aptamer biosensors is becoming increasingly prevalent. QDs are nanocrystals and are usually comprised of atoms from elemental groups II and VI (e.g. cadmium sulfide and cadmium selenide) or groups III and V (e.g. indium phosphide and indium arsenide) [54]. The small radii (in the nanometer scale) of these structures lead to electron confinement, giving rise to their unique optical and electronic properties. While the absorption spectrum of QDs is broad, their emission spectrum is narrow and is tunable by adjusting their size. In order to enhance their quantum yield and produce brighter signals, a surface capping layer, such as a zinc sulfide shell, may be deposited on their surfaces. Moreover, the use QDs as signal transducers in place of fluorophores has an additional advantage owing to their resistance toward photobleaching.

**Figure 7.**
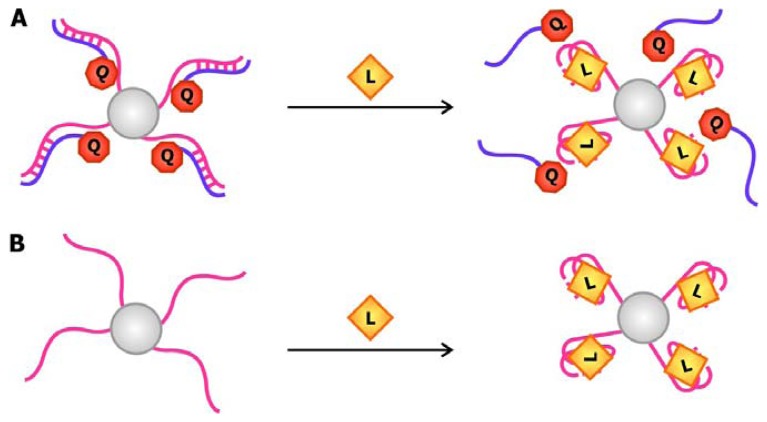
Quantum dot (QD)-based biosensors. a) The FRET-based QD aptamer biosensor by the Ellington group [55]. b) In the QD-based biosensor by Choi and colleagues [56], target binding results in the transfer of a charge from the ligand to the QD, resulting in the quenching of its photoluminescence.

Earlier in 2005, the Ellington group created a FRET-based QD aptamer biosensor in which the QD is decorated with multiple thrombin-binding aptamers (Figure 7a) [55]. These aptamer sequences are bound by a quencher-modified antisense sequence in the absence of thrombin, thus the signal emitted from the QD is dampened. The binding of thrombin causes the aptamer to fold up, releasing the quencher-modified strand as well as the signal from the quantum dot. In another sensor reported by Choi *et. al.*, the QDs are capped with the shorter variant of thrombin aptamers (Figure 7b) [56]. The capping of nanocrystals with aptamers does not only allow for the recognition of biomolecules, they also improve their water solubility by preventing colloidal aggregation. In this construct, the thrombin bound to the aptamer transfers a charge to the QD, such that target binding is signaled by quenching of the QD's photoluminescence as a result of this charge transfer.

## 8. Immobilized acoustic sensors- orchestrating the signal from binding

**Figure 8.**
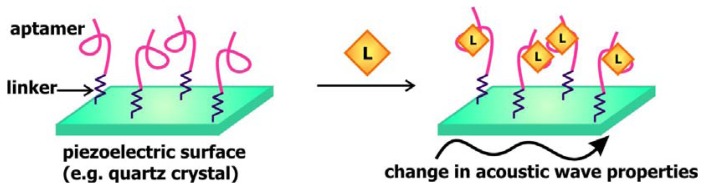
General design and mechanism of acoustic aptamer biosensors [57].

In a manner that is comparable to signal transduction by SPR-based biosensors, acoustic sensors utilizes the change in the properties of waves traveling along surface of the signal transducer to communicate the presence of targets. In these sensors, a piezoelectric material, such as quartz crystals, is used as the signal transducer (Figure 8). These surfaces are frequently coated with gold and modified with streptavidin or a self-assembled monolayer of linkers to allow for the association of aptamers to these chips. Mass loading resulting from target binding to these aptamers decreases the velocity of surface acoustic waves propagating along the signal transducer, consequently decreasing the resonance frequency of these waves or changing the phase shift between input and output waves [57]. Thus, monitoring the change in one of these two properties would provide an indication of the absence or presence of the ligand of interest.

Schlensog and colleagues have previously developed acoustic aptamer biosensors that signal through Love waves, which are a form of horizontally propagating surface acoustic waves [57]. This group coupled single-stranded DNA sequences as well as aptamers against human thrombin and HIV-1 Rev proteins onto a gold layer atop a quartz crystal. The binding of thrombin and Rev to their corresponding aptamers and the hybridization of DNA sequences complementary to the immobilized ss-DNA would result in a detectable phase shift of the Love wave. The sensors developed are capable of accurately detecting their relevant target with a low detection limit of 70 to 80 pg/cm^2^. Gronewold and colleagues later demonstrated that the Love wave-based thrombin biosensor may be used to monitor protein-protein interactions and complex formation [58].

The use of quartz crystals enables an alternative approach of acoustic sensing. Mass loading on the surface of the sensor can alter the natural frequency of the resonance wave propagated along the quartz crystal resonator, and this frequency shift may be measured by a quartz crystal microbalance (QCM). Mascini's group engineered a QCM-based aptamer biosensor comprised of aptamers specific for the HIV-1 Tat protein [45]. Comparing this sensor with an SPR-based Tat aptamer sensor, they found that the sensitivity, reproducibility, and specificity of the two sensors are analogous. Recently, this group generated a QCM-based aptamer biosensor for thrombin detection and demonstrated that this sensor can be reused up to twenty times without diminishing its sensitivity [59].

## 9. Micromechanical sensors- may the force be with you

An alternative label-free target detection approach involves the use of microcantilevers as signal transducers. When forces, such as the forces associated with mass loading, act on the surface of cantilevers, the cantilever will bend or deflect [60]. Thus, target recognition may be evaluated by measuring the extent of cantilever deflection or changes in resonance frequency, force constant, and specific heat capacity (ΔQ) in the material upon mass loading.

**Figure 9.**
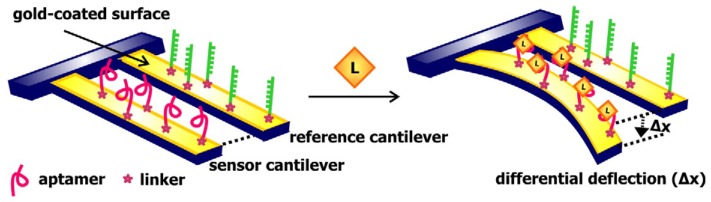
Design and mechanism of cantilever-based aptamer biosensors by Savran and colleagues [62]. Using this system, ligand concentration may be assessed by measuring the extent of deflection of the sensor cantilever.

The use of sensors consisting of nucleic acids as recognition elements and microcantilevers as signal transducers was first realized in 2000 when Fritz and colleagues synthesized a sequence-specific sensor for complementary oligonucleotides by immobilizing ss-DNA onto a microfabricated silicon cantilever array [61]. Cantilevers used with nucleic acids are typically coated with gold in order to permit the covalent linkage of sequences modified with a thiol group on one end to the surface of the array. Later, in 2004, Savran and colleagues synthesized a cantilever-based aptamer biosensor, which makes use of two cantilevers connected by a solid support (Figure 9) [62]. One of the cantilevers, which serves as the sensor, was functionalized with thiol-modified aptamers specific for *taq* polymerase. The second cantilever, functionalized with single-stranded nonspecific DNA sequences, serves as the reference. The binding of the *taq* polymerase to the aptamers causes the sensor cantilever to bend. The extent of bending depends on the ligand concentration and can be measured using the optical lever technique. This technique measures the change in location of a beam of light reflected from a laser beam focused on the tip of the cantilever as a result of mass loading and cantilever deflection. To minimize the effects of non-specific binding and other disturbances, the signal was reported as differential deflection, which is determined by subtracting the deflection of the reference cantilever from the deflection of the sensor cantilever. The advantages of using cantilevers as signal transducers were further illustrated when Huber and colleagues linked double-stranded DNA sequences containing specific binding sites for two different transcription factors on a microcantilevers array and showed that these transducers can report multiple bimolecular interactions in parallel [63].

## 10. Electronic sensors- they have potential

In recent years we are seeing the emergence and growth of a collection of aptamer biosensors that signal by electronic or electrochemical means. Compared to the array of optical aptamer biosensors that are at our disposal, these biosensors are more cost-effective, more stable, less susceptible to contaminants and non-specific binding, and involves the use of simpler instrumentations for detection [64]. Furthermore, the detection of electrical signals is not affected by factors such as solution turbidity or opacity, which may cloud the detection of fluorescence signals. Like their optical predecessors, electronic biosensors with diverse signaling approaches are available. A comprehensive overview of the progress in the development of electronic aptamer biosensors is presented in a review by Willner and Zayats [65]. Here, we will briefly discuss the designs, mechanisms of signaling, and potential applications of some of these sensors.

**Figure 10.**
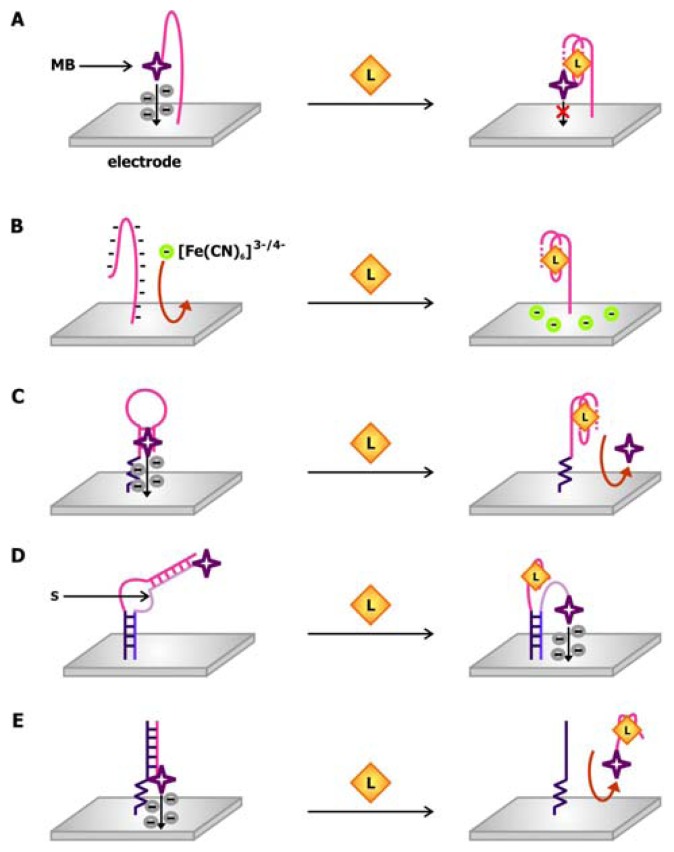
Examples of electronic aptamer biosensors. a) A “signal-off” thrombin biosensor that uses methylene blue (MB) as a redox indicator [64]. b) A *trans*-signaling biosensor that depends on the interaction between [Fe(CN)_6_]^3-/4-^, a negatively-charged redox indicator, and the electrode [68]. c) A “signal off” thrombin sensor that exploits methylene blue's to function as both a DNA intercalating molecule and a redox indicator [69]. d) A structure-switching electronic aptamer biosensor in which the aptamer is bound by a redox dye-modified signaling oligonucleotide (S) in the absence of ligand [70]. Ligand binding displaces the oligonucleotide from the aptamer, allowing the dye to contact the electrode and generate a current. e) An electronic biosensor that signals by strand release [72].

The design of one of the earlier electrochemical aptamer biosensors is mimetic of the molecular beacon in which the aptamer is attached to two signaling moieties and the signal produced by the sensor is dependent on the structural changes in the aptamer following ligand binding. In a thrombin biosensor engineered by the Plaxco group (Figure 10a), the thrombin aptamer is attached to a gold electrode via its 5′ end and linked to methylene blue via its 3′ end [64]. Methylene blue, which may be used as a stain for nucleic acids owning to its ability to intercalate DNA and RNA, is used a redox indicator in this sensor. In the absence of thrombin, the aptamer adopts a flexible, unfolded configuration, promoting electron transfer from methylene blue to the electrode when the two components collide. In the presence of thrombin, the aptamer is driven to fold into its binding-competent guanine-quartet conformation, which in turn prohibits electron transfer due to its rigidity.

In contrast to this “signal off” sensor, which makes use of a 32-nt thrombin aptamer sequence, the O'Sullivan group produced a “signal on” thrombin biosensors using a 15-nt aptamer sequence [66]. In this sensor, the aptamer immobilized on the gold electrode is modified with ferrocene at its 3′ end. Since this aptamer is shorter in length, the structured, ligand-bound conformation brings ferrocene closer to the electrode, thereby favoring the collision and electron transfer between them. Both of these sensors can detect thrombin in the nanomolar range and can produce a response within minutes. The latter sensor can be washed with 1M HCl following thrombin detection, so that it can be reused for up to 25 times without triggering a loss in activity.

Using this electrochemical molecular beacon approach, the Plaxco group developed a “signal on” cocaine biosensor comprised of a cocaine aptamer connecting an electrode to methylene blue [67]. In this system, cocaine binding induces folding of the aptamer into a three-way junction structure, thus decreasing the distance between the methylene blue dye and the surface of the electrode and, as a result, the resistance of electron transfer. Although this sensor can generate a response within seconds, its detection limit is only in the micromolar range. Nonetheless, once the sensitivity of this cocaine biosensor is optimized, it will prove to be an asset in law enforcement and in clinical settings considering that it that can detect the compound in blood serum, saliva and other biological samples.

To circumvent the need to covalently link the redox indicator to the aptamer, it may be administered *in trans* to the aptamer-linked electrode in order to produce a signal. The aptamer biosensor presented by Rodriguez and colleagues relies on the change in the surface charge of the electrode upon the binding of a protein with a large isoelectric point (pI) [68]. In this sensor, an indium oxide electrode is functionalized with a lysozyme-binding aptamer (Figure 10b). Since the backbone of the nucleic acid aptamer is negatively charged, it repels the binding of [Fe(CN)_6_]^3-/4-^, a redox indicator, to the electrode and hinders subsequent interfacial electron transfer. Following the binding of lysozyme, which has a pI of 11, an excess positive charge is deposited on the electrode and the repulsion against [Fe(CN)_6_]^3-/4-^ is alleviated.

As mentioned previously, in addition to being a redox indicator, methylene blue is also a DNA intercalating dye. This hybrid signaling capability is exploited in the thrombin sensor conceived by Bang and colleagues [69]. As illustrated in Figure 10c, the 22-nt thrombin aptamer employed in this sensor is modified with a primary amine at its 5′ end allowing it to be linked to a gold electrode. When it is freed from thrombin, it forms a hairpin structure that may be intercalated by methylene blue. Thrombin binding promotes the formation of the rigid ligand-binding guanine quartet, thus disrupting the hairpin. Consequently, methylene blue dissociates from the aptamer, and the electrochemical signaling arising from its electron transfer to the electrode is reduced.

Combining the molecular beacon and *trans*-acting signal approaches, Xiao and colleagues developed structure-switching biosensors in which the unmodified aptamer is connected to the electrode via an oligonucleotide linker (Figure 10d) [70]. A signaling oligonucleotide, which is complementary to both the linker and the aptamer sequences on the sensing oligonucleotide, is modified with a redox marker and attached to the electrode. These two sequences hybridize in the absence of target, causing the signaling oligonucleotide to be more rigid. Due to the distance between the redox dye and the electrode, resistance against electron transfer is increased. The presence of the target displaces the redox dye-bound segment of the signaling oligonucleotide from the sensing oligonucleotide in order to accommodate target binding. This renders the signaling oligonucleotide more flexible and accessible to the electrode surface, generating a current in the electrode. Using this strand displacement approach, Xiao and colleagues created a thrombin aptamer-binding biosensor with a detection limit in the low nanomolar range [70]. Later, Lai *et al.* produced a sensor for complementary oligonucleotides with an impressive detection limit in the femtomolar range [71], consequently showcasing the potential power of these electrochemical biosensors.

To take the strand displacement technique one step further, Yoshizumi and colleagues developed an aptamer biosensor that signals by strand release (Figure 7e). In this sensor, the dye-modified aptamer is not directly attached to the electrode [72]. Rather, the electrode is functionalized with a DNA sequence that is complementary to the aptamer sequence. Therefore, when the aptamer is not bound by its target, it will hybridize with the complementary DNA (cDNA), so that methylene blue would be brought to the proximity of the electrode. The hybridization is disrupted when the aptamer associates with its target and this duplex-to-complex structural change is reported by a decrease in the electrochemical signal. This target-induced strand release approach of signaling was examined using aptamers against ATP as well as against thrombin, thus demonstrating that this approach is amenable to both small molecules and macromolecules. This mechanism was also applied in the “signal on” ATP and thrombin sensors conceptualized by Lu and colleagues [73]. In this sensor, a redox indicator-modified cDNA is immobilized on the electrode and forms a duplex with an unmodified aptamer when the target is scarce. When it is abundant, however, it associates with the aptamer, forcing the aptamer to be displaced from the cDNA. Subsequent heating of this electrode-associated, single-stranded cDNA in the presence of magnesium favors its folding into a hairpin stem that pushes the redox dye closer to the electrode surface, producing a signal. Despite the differences in the design and mechanisms of signal production in the sensors by Yoshizumi and Lu, both types of sensors share comparable sensitivities and exhibit detection limits in the nanomolar range.

Amalgamating duplex-to-complex structure-switching with the use of ion selective field effect transistors (ISFET), the Willner group introduced a novel type of label-free electronic aptamer biosensor [74]. In this sensor, ATP-targeting aptamers are immobilized on the gate surface of an ISFET device, forming a sensing surface. This gate is deposited on a semiconductor, which separates a source electrode from a drain electrode. In order to enable the detection of changes in gate potential arising from target recognition, this device is submerged in an analyte solution and the source electrode is connected to a reference electrode. In its ligand-free state, the aptamer is bound by a complementary oligonucleotide. However, this anionic oligonucleotide is liberated from the aptamer upon ATP binding, subsequently changing the charge of the gate and, consequently, the current running from the source electrode to the drain electrode. To maintain a constant current between these two electrodes during the structure-switching event, the electric potential between the source and reference electrodes may be adjusted to control fluctuations in gate potential. Hence, ligand binding may be detected by measuring the change in potential between the source and reference electrodes. More recently, this group incorporated the use of a bifunctional aptamer, which can simultaneously detect cocaine and AMP, with ISFET-based sensors and demonstrated the possibility of using aptamer biosensors for multiplexed sensing [75]. In addition to the ISFET-based biosensor, the Lee group has developed a thrombin-sensing FET-based aptamer biosensor using single-walled carbon nanotubes (SWCNT) as the gate [76]. Although both the SWCNT-FET and ISFET-based aptamer biosensors are approximately 10-fold less sensitive than some of the aforementioned electronic biosensors, their preparation is more facile compared with the other sensors as they do not require external labels.

## 11. Passing the signal- FNAs assist aptamer biosensing

The integration of aptamers with other FNAs to produce functional sequences with small molecule, metabolite, metal ion or protein-dependent activities was introduced to us toward the end of the past century. While this concept of producing allosteric FNAs by rational design was novel to us, Nature has beaten us to the punch and has long practiced this technique, as proven by the presence of metabolite or metal ion-binding riboswitches, allosteric ribozymes, and protein-mediated non-coding RNAs in the cell. It is anticipated that a plethora of these naturally-occurring allosteric FNAs are still waiting to be discovered. As we are waiting for these sequences to surface, a series of synthetic sequences have been created, including ones that cannot or are not known to exist in the cell.

**Figure 11.**
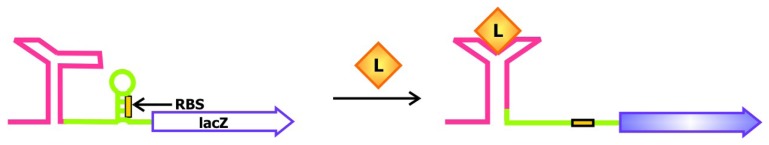
Design of a theophylline sensor based on the use of the artificial theophylline riboswitch, which regulates the translation of the *lacZ* reporter gene [19].

Applying modular rational design, Breaker and his colleagues previously generated ribozymes with ATP, FMN or theophylline-regulated activities [77, 78]. This was accomplished by joining aptamers with the desired ligand specificity to a self-cleaving ribozyme via a bridge element, which has been optimized by *in vitro* selection. In this tripartite system, the energy derived from ligand binding drives the conformational change in the bridge and catalytic regions, subsequently activating or inhibiting the activity of the ribozyme by more than 100-fold [78, 79]. Adopting this concept of modular design, allosteric FNAs that can function as sensors or genetic control elements may be engineered. For example, Suess and colleagues reported the engineering of an artificial theophylline riboswitch in which the theophylline aptamer is connected to the open reading frame of a *lacZ* reporter gene through a helical communication module (Figure 11) [19]. In the absence of theophylline, the ribosome binding site (RBS) of the gene is sequestered and *lacZ* expression is supressed. Upon ligand binding, a single nucleotide slippage is elicited in the communication module and the RBS is alleviated. This permits the ribosome to access the mRNA and initiate translation to synthesize β-galactosidase, an enzyme which can metabolize 5-bromo-4-chloro-3-indolyl-β-D-galactopyranoside (X-gal) to produce a product that is ultimately oxidized to form a blue-coloured compound known as 5,5′-dibromo-4,4′-dichloro-indigo. By assaying the production of this compound, *lacZ* expression may be evaluated. It was demonstrated that this artificial riboswitch retains its function inside the cell. Therefore, intracellular levels of theophylline in cells transformed with these constructs can be measured through simple *in vivo* assays of β -galactosidase activity. In an alternative system designed by the Gallivan team, the theophylline aptamer was appended upstream of a gene along the chemotactic signal transduction pathway, such that the expression of the gene is theophylline inducible [80]. Monitoring the motility of cells carrying these constructs may be used to detect theophylline in the growth media. The regulatory function of both of these theophylline-based riboswitches presents the possibility of building biosensors with synthetic or naturally-occurring riboswitches as the MRE and the riboswitch-regulated gene as the signal transducer.

**Figure 12.**
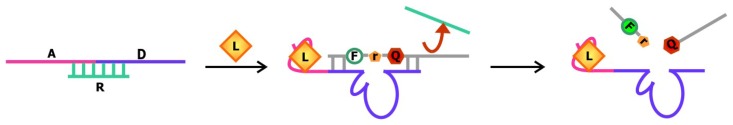
Aptazymes- using aptamer-deoxyribozyme constructs as biosensors [81]. In this system, ligand binding to the aptamer (A) displaces the regulatory oligonucleotide (R) that binds with both the aptamer and the deoxyribozyme (D). This allows the deoxyribozyme to interact with and cleave its chimeric DNA-RNA substrate in order to produce a fluorescence signal.

Rational design was also behind the development of aptamer biosensors that depend on the activity of deoxyribozymes to produce a signal. Previously, the Li group has engineered a series of sensors using RNA-cleaving deoxyribozymes. In one system, an aptamer is appended onto the deoxyribozyme [81]. When the target is not bound, the aptamer is unfolded and a complementary regulatory oligonucleotide can bind to part of the aptamer and part of the deoxyribozyme sequence and prohibit the interaction between the deoxyribozyme and its substrate (Figure 12). Target binding induces a conformational change in the sequence, which liberates the deoxyribozyme from the regulatory sequence and favors substrate cleavage. In another system, the aptamer is sandwiched by the substrate and the regulatory sequence [82]. The aptamer and regulatory oligonucleotide fold back to block part of the substrate from being accessed by the deoxyribozyme in its unbound state. When the target is introduced, alternative folding in the aptamer is induced, freeing up the substrate and allowing it to engage the deoxyribozyme. In both of these allosteric deoxyribozyme systems, the signal for target detection arises from the design of the chimeric substrate in which the single ribonucleotide at the cleavage site is flanked by nucleotides modified with a fluorophore and a quencher on each side. This way, the fluorescence signal emanating from these constructs remains low until the sequence is cleaved. Currently, the Li group is working to improve the detection limit and sensitivity of existing biosensors by exploring deoxyribozymes with different ion specificities.

**Figure 13.**
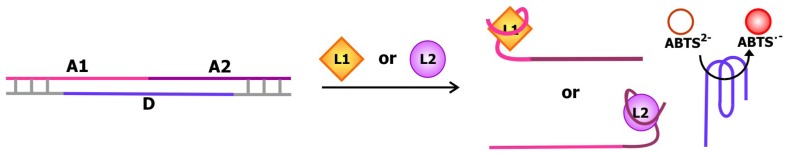
A structure-switching biosensing system comprised of the bifunctional aptamer that can bind either ligands L1 or L2 and a deoxyribozyme with horseradish peroxidase-like activity (D) [75]. The binding of either L1 or L2 results in the liberation of the deoxyribozyme, allowing it to oxidize ABTS^2-^ to produce the red-coloured ABTS^-^.

More recently, the Willner group created a structure-switching biosensing system comprised of the bifunctional cocaine and AMP-specific aptamer described in the previous section and a deoxyribozyme with horseradish peroxidase-like activity [75]. The deoxyribozyme is flanked by sequences that are complementary part of the aptamer on each side as depicted in Figure 13. The aptamer-deoxyribozyme duplex that is formed in the absence of cocaine or AMP is more stable than the self-assembled guanine quartet in the DNA enzyme. Thus, the enzyme is sequestered. Cocaine and/or AMP binding stimulates the formation of the ligand-aptamer complex and the release of the deoxyribozyme. This way, the deoxyribozyme can catalyze the hydrogen peroxide-mediate oxidation of 2,2′-azino-bis(3-ethylbenzothiazoline)-6-sulfonic acid (ABTS^2-^) to produce ABTS^.-^, a colored product. Using this system, the concentrations of cocaine and AMP may be measured by assaying the activity of the DNA enzyme.

## 12. Aptamer bioassays- applying aptamers in research

Since the conception of the first aptamers in 1990, we have seen the creation of hundreds of target-binding DNA and RNA sequences, each with unique ligand specificity. Moreover, our collection of aptamer-based biosensors is expanding. For certain targets, we can select sensors with signaling motifs that best suit our needs and convenience. Despite this impressive progress, however, we now need to go beyond the stage of simply demonstrating that we can synthesize aptamers by different approaches of *in vitro* selection and aptamer-based biosensors by conjugating aptamers with various signal transducers. We need to progress to finding experimentally and clinically relevant applications for these aptamer-based tools.

**Figure 14.**
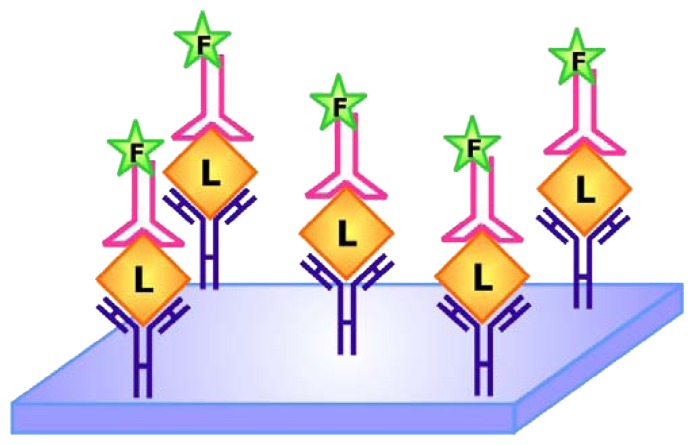
ELONA (Enzyme-linked oligonucleotide assay) is a bioassay that makes use of antibodies to capture a ligand of interest and fluorescently-labeled aptamers as a reporter for target detection [84].

Since its conceptualization in 1971, enzyme-linked immunosorbent assay (ELISA) has been widely used in research and in diagnostic medicine [83]. One approach of ELISA, commonly referred to as sandwich ELISA, involves the simultaneous use of two antibodies or analyte-binding receptor proteins to capture the analyte of interest and to report target detection. Following this idea of sandwich ELISAs, Drolet and colleagues developed a new detection method that is appropriately coined ELONA (enzyme-linked oligonucleotide assay) in which the reporting antibody/ protein of ELISA is substituted for a fluorescein-tagged aptamer specific for detecting the target of interest (Figure 14) [84]. Using an aptamer against human VEGF as a model, Drolet *et al.* showed that ELONA may be applied to quantify hVEGF in sera and its accuracy and specificity are comparable to that of the well-established ELISA. As mentioned earlier in the review, aptamers tend to have a longer shelf-life and are easier to synthesize compared with antibodies. Considering that aptamers are approximately 10 times smaller than antibodies, they are also more facile to modify and label. These advantages render the use of ELONA for analyte detection even more appealing than using ELISA for similar purposes.

To further exclude the use of antibodies in sandwich assays, Vivekananda and Kiel devised a method that employs aptamers as the target capturing and reporting elements [85]. This technique was coined ALISA (aptamer-linked immobilized sorbent assay) and was applied by Vivekananda and Kiel to examine the specificity of an aptamer against an antigen associated with *Francisella tularenis*, a bacteria which causes tularemia or rabbit fever, an infectious endemic disease.

Aptamers can not only substitute antibodies in existing analyte detection techniques, they can also replace proteins in analyte separation techniques. As described in a review by the Mascini group, aptamers have been used as affinity probes in chromatography and in capillary electrophoresis [86]. For example, Murphy and colleagues previously coupled biotinylated aptamers for thyroid transcription factor 1 (TTF1) onto streptavidin-coated magnetic beads in a column and used the column to isolate TTF1 from cell lysates by affinity chromatography [87]. Using aptamers specific for D-adenosine and L-tyrosinamide, Michaud and coworkers further revealed that aptamers may be used as enantioselective resins in high performance liquid chromatography (HPLC) [88].

After capturing the analytes of interest with aptamers or aptamer-based biosensors, the analyte and its associated molecules may be further characterized. As demonstrated in a proof-of-concept study by Treitz *et. al.*, human α-thrombin and its binding partner, human antithrombin III, may be detected by an acoustic biosensor built with aptamers against human α-thrombin [89]. After digesting the bound proteins with proteases, matrix-assisted laser desorption/ ionization time-of-flight (MALDI-TOF) mass spectrometry may be applied to produce peptide mass fingerprints in order to identify the proteins. To characterize the proteins individually, the proteins in the complex may be separated by nano-capillary HPLC prior the being subjected to MALDI-TOF mass spectrometry. Once this system is implemented with more aptamers, it is hopeful that it can be developed into a technique for proteomic studies or for diagnostic medicine. For example, it may be applicable for examining protein-protein interactions in cellular pathways or for identifying aberrant protein associations in diseased states.

## 13. Applying aptamers in diagnostics, therapeutics, and drug discovery

Research has already been initiated in developing aptamer-based biosensors to detect cytotoxins and disease markers. Back in 2000, Ellington's laboratory has published a report regarding the isolation of an aptamer specific for ricin, a popular toxin for biological warfare that inhibits protein translation in cells and causes severe respiratory distress in its hosts [90]. The aptamer isolated by the Ellington group binds the catalytic ricin chain A with a high affinity (K_d_= 7.3 nM) and can compete with its natural substrate. This aptamer can also serve as an inhibitor of ricin. With an IC_50_ that is approximately three orders of magnitude lower than pteroic acid, a small molecule ricin inhibitor, this aptamer provides us with powerful ammunition against biowarfare. Immobilizing this aptamer onto an electronic taste chip, the Ellington group produced an aptamer sensor array that can be used to capture and detect ricin via a sandwich assay [91]. A ricin-specific antibody was employed to detect the chip-bound ricin. This reusable chip is capable of detecting unlabeled ricin at a concentration that is as low as 320 ng/mL and it may be applied to quantify the toxin [92]. Recently, Tang and colleagues isolated an aptamer against aubrin, a class II ribosome inactivating protein, and converted it into an optical biosensor using [Ru(phen)_2_(dppz)]^2+^, which is a molecule that emits a strong luminescence signal only when bound to duplex nucleic acids, such as the folded, ligand-associated form of the aubrin aptamer. This biosensor was shown to be highly selective and displays a detection limit of 1 nM. In the past, aptamers against other commonly used biowarfare agents, such as anthrax spores, cholera toxin and staphylococcal enterotoxin B, have been reported by Bruno and Kiel [93, 94]. Once the sensitivity and selectivity of these aptamers are improved, it is optimistic that they too may be converted into biosensors.

The toxins described above are agents that we might encounter under extreme and unusual circumstances. In our everyday life, we are constantly bombarded with other compounds that can jeopardize our well-being. Currently, a series of aptamers have already been isolated for the detection of some of these compounds. For example, Brockstedt and colleagues have selected for an aptamer against 4,4′-methylenedianiline (MDA), a potential carcinogen that might be used as an industrial chemical for the manufacturing of plastics, glues and polyurethane foams [95]. The Lu group developed biosensors for lead (Pb^2+^), a toxic metal that may cause blood disorders or damage the nervous system, especially in young children. These sensors make use of a lead-dependent deoxyribozyme for target detection. In one scenario, the signal is emanated from a change in fluorescence associated with the cleavage of a fluorophore and quencher-modified chimeric substrate by the enzyme in the presence of lead [96]. In another system, the enzyme and substrate are connected to gold nanoparticles, and the lead-dependent substrate cleavage is signaled by the color change associated with the aggregation states of these AuNPs [97, 98].

Similar to toxins, infectious particles are also agents that can impact our cells and cause serious illnesses. Recently, a system was established for detecting a proteinaceous form of these particles, known as prions, which are associated with neurological diseases, such as transmissible spongiform encephalopathies [99]. Although the exact nature of prions remains obscure, they are proposed to be infectious misfolded proteins. Previously an aptamer against the normal, cell membrane bound form of prions (Prp^c^) was isolated [100]. As far as we currently understand, the Prp^c^ form of prions may be converted to its infectious isoform, Prp^sc^, simply by coming into contact with Prp^sc^. Functionalizing the biotinylated version of this aptamer onto streptavidin-coated magnetic beads or gold nanoparticles, Kouass and researchers developed a prion biosensor. Binding of Prp^c^ to the sensor may be determined by Fourier-transform infrared spectroscopy.

An aptamer isolated by Pan and colleagues was proven to bind the IVB pili of *Salmonella enteric* serovar *typhi*, a typhoid and enteric fever-causing pathogen that is frequently transmitted through contaminated food and water [101]. The pilus of the organism facilitates its pathogenic process, enabling its adhesion and invasion of human gastrointestinal epithelial cells. When it is aptamer-bound, however, its entry into cells belonging to the human monocytic leukemia cell line is inhibited. This finding suggests that the dual function of this aptamer allows it to be modified into tools for detection as well as for prophylactic purposes.

In addition to infections, aptamer biosensors for the detection of disease markers are now among the list of aptamer-based tools with clinical applications. An optical sensor recently published by the Mascini group was reported to be capable of sensing C-reactive protein (CRP), a clinical biomarker for inflammation and tissue damage [102]. An elevated level of this protein is also indicative of acute coronary syndrome. In normal adults, the average concentration of CRP is around 0.8 ppm. However, in response to inflammation stimuli, including those associated with myocardial infarctions, serum concentration of CRP may increase to as high as 500 ppm. The sensor by Mascini and his team has a detection limit of 0.005 ppm and a linear range of 0 to 0.1 ppm or 0 to 0.5 ppm depending on the concentration of aptamers used as well as the linker attached to the aptamer for immobilization purposes. Through this preliminary study, the conditions required for the optimal function of this biosensor, such as buffer pH and ion concentration, was evaluated. In another study by Lee and colleagues, a DNA aptamer specific for retinol binding protein 4 (RBP4), a biomarker for insulin resistance and type II diabetes, was converted into an SPR biosensor after being functionalized onto a gold chip [103]. When tested with artificial serum, the sensitivity, dose-dependent response, and speed of detection of this aptamer-based biosensor rival that of existing antibody-based methods for RBP4 detection, such as Western blotting and ELISA. This biosensor also has the additional advantage of being reusable and thermal stable.

As we have discussed, aptamer-based biosensors may be applied in disease detection and prevention. When detection and prevention are insufficient to defend against infections and other maladies, treatment is our best option in sustaining our well-being. Naturally-occurring aptamers that reside within riboswitches have already been examined as potential and novel targets for small molecule inhibitors or activators predominantly by the Breaker group. They found that pyrithiamine, an analog of thiamine, can bind the pyrophosphate thiamine (TPP) riboswitch with high affinity and suppress the expression of the downstream gene [104]. Likewise, *S*-(2-aminoethyl)-L-cysteine (AEC) can tightly bind the lysine riboswitch and suppress the expression of the riboswitch-regulated *lysC* gene *in vivo* [105]. Since *lysC* encodes a precursor for lysine biosynthesis as well as for bacterial cell wall biosynthesis, AEC is proposed to have antimicrobial action. The Breaker group and the Famulok group separately carried out high throughput screens with FRET-based and fluorescence-based assays respectively in an effort to isolate compounds that would bind the *glmS* riboswitch and stimulate the self-cleavage of mRNA [106, 107]. It was reported that this self-cleavage destabilizes the mRNA, thereby preventing its translation to produce glucosamine-6-phosphate synthetase, which is responsible for the biosynthesis of another bacterial cell wall precursor known as glucosamine-6-phosphate. Therefore, the small molecule activators isolated from these screens also have the potential of being riboswitch-targeting antimicrobials.

A recent review by Srivatsan and Famulok accounts for various efforts in integrating aptamer biosensors with HTS to isolate compounds that can interfere with cellular processes and hinder cell growth or elicit cell death in the hopes of generating novel antibiotics and other therapeutics [108]. One of these screens was conducted by Elowe and colleagues [109]. Using a structure-switching signaling aptamer specific for adenosine, a library consisting of 44 000 compounds was screened via rapid and robust fluorescence assays in search of an inhibitor targeting adenosine deaminase (ADA), a crucial enzyme in purine metabolic pathways. Specifically, it catalyzes the conversion of adenosine/ deoxyadenosine to inosine/ deoxyinosine. Since the aptamer used for the screen selectively binds adenosine but not inosine, the presence of an ADA inhibitor would contribute to an elevated level of adenosine, as indicated by an increase of fluorescence signal. The success of this particular screen showcases the possibility of using aptamers biosensors that are specific for the substrate or the product of any enzyme to identify its inhibitor.

## 14. Conclusions

Since its emergence in 1990, the field of aptamer research has flourished tremendously. *In vitro* selection has undoubtedly brought this field to fruition, providing hundreds of aptamers, each with a unique ligand specificity, at our fingertips. These aptamers can bind substances ranging from organic dyes to small metabolites to proteins. While the isolation of aptamers with novel ligand specificities remains exciting, current attention is focused on the discovery of aptamers against targets with biological or clinical relevance, such as cytotoxins, infectious agents and disease markers. Findings in this avenue of aptamer research will not only emphasize the extraordinary ability of aptamers to fold into distinct three-dimensional constructs and interact with selected ligands, they will also showcase the utility and practicality of aptamer research. We have already witnessed the potential of using aptamers against certain protein enzymes as inhibitors and the possibility of developing aptamer-based therapeutics, as demonstrated by the commercially-available, FDA-approved drug, Macugen, and other anticoagulation, anti-inflammatory, and anti-proliferative drugs that are in clinical trials [26, 110, 111]. Archemix, a company specializing in the development of aptamer-based therapeutics, has developed anticancer aptamers that have been shown to be active in patients with renal carcinoma and acute myeloid leukemia in phase one and phase two clinical trials, respectively (www.archemix.com). The engineering of aptamer-based diagnostic and research tools is also gaining prominence. Several groups have independently shown that aptamers can substitute for the use of antibodies in well-established separation techniques, bioassays, and diagnostic tools. We are also seeing the commercialization of some of these aptamer-based technologies. For example, SomaLogic manufactures platforms containing aptamer arrays that enables for simultaneous detection of multiple biomarkers (www.somalogic.com). The development in aptamer biosensors, the spotlight of this review, further validates that aptamers can easily be integrated in many practical applications. The anatomy of these biosensors is quite basic- they may be created simply by joining an existing aptamer, which serves as the molecular recognition element, to a signal transducer. Yet, this modular design is used to create various prototypical aptamer biosensors, whether they signal by optical, acoustic, mechanical, or electrical means.

Although we now have a vast selection of aptamer biosensors with different target specificities and rapid signaling capabilities at our disposal, the full potential of these biosensors have not yet been realized. The number of targets that can be recognized by our current library of aptamers is meager relative to the multitude of endogenous, as well as exogenous metabolites, macromolecules, and other biologicially-relevant substances that we might wish to detect. This limitation emphasizes the need to conduct more selection studies to expand the population of ligands that are recognizable by aptamers. This process may be facilitated by the advent in the development of automated SELEX. In order for these biosensors to work at physiologically-relevant settings, we need to establish adaptable sensors that will retain their optimal functions under a broad range of environments. For example, the conformation and specificity of aptamers are often dependent on ionic strength, because cations can reduce the electrostatic forces in the DNA backbone. As such, many aptamer-based biosensors may only exhibit their optimal response under specific ionic environments [112]. It is therefore beneficial to engineer sensors that will be functional under desired conditions or develop ones that will retain their response and sensitivity under diverse conditions. Further work will also have to be done to fine-tune the detection limit and dynamic range of existing sensors, so that they can detect targets at physiologically-relevant concentrations. As it was alluded to earlier, several biosensors are shown to be capable of detecting their cognate ligands in complex mixtures such as biological fluids, suggesting that with the proper delivery systems, these sensors may be applied *in vivo*. In the past two years, several groups have reported the immobilization of aptamers and aptamer biosensors onto materials such as cellulose [113], microgels [114] and xerogels [115]. This paves the way for the production of sensors that are portable and easy to use.

The development of aptamer-based biosensors is continuing to progress at a rapid rate. It is hopeful that in the near future, the field will advance to the point where we are able to custom-design these biosensors on demand and choose aptamers and signal transducers that best suit our purpose and convenience.

## Figures and Tables

**Table 1.** Design strategies of fluorescence signaling aptamer biosensors.

**Design**	**Selected Examples**
**A. Signaling by a single fluorophore** 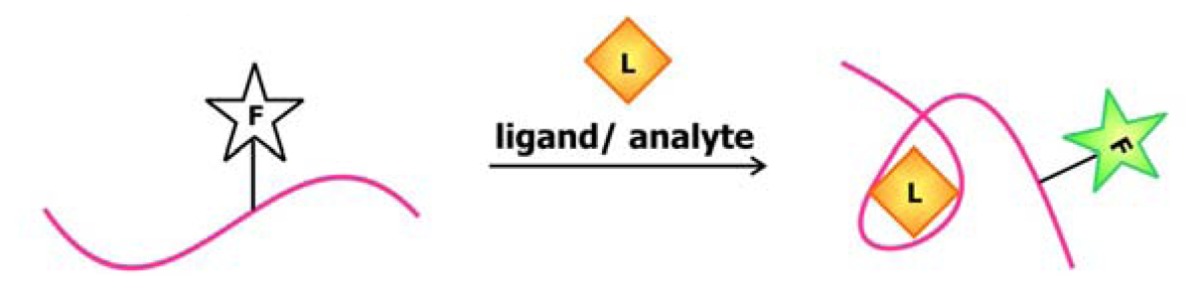 To produce an aptamer biosensor with a single fluorophore signal transducer, a fluorophore may be covalently linked to the aptamer or a fluorescent nucleotide analogue may be incorporated into the sequence at a position that is expected to undergo significant structural reorganization following ligand (L) binding. In this system, target-aptamer interaction is reported by either an increase or a decrease in fluorescence intensity resulting from the change in the electronic environment surrounding the fluorophore upon ligand binding.	ATP sensor [31, 32]; Thrombin, Immunoglobin E, PDGF-β sensors [33]
**B. Signaling by a fluorophore-quencher pair** 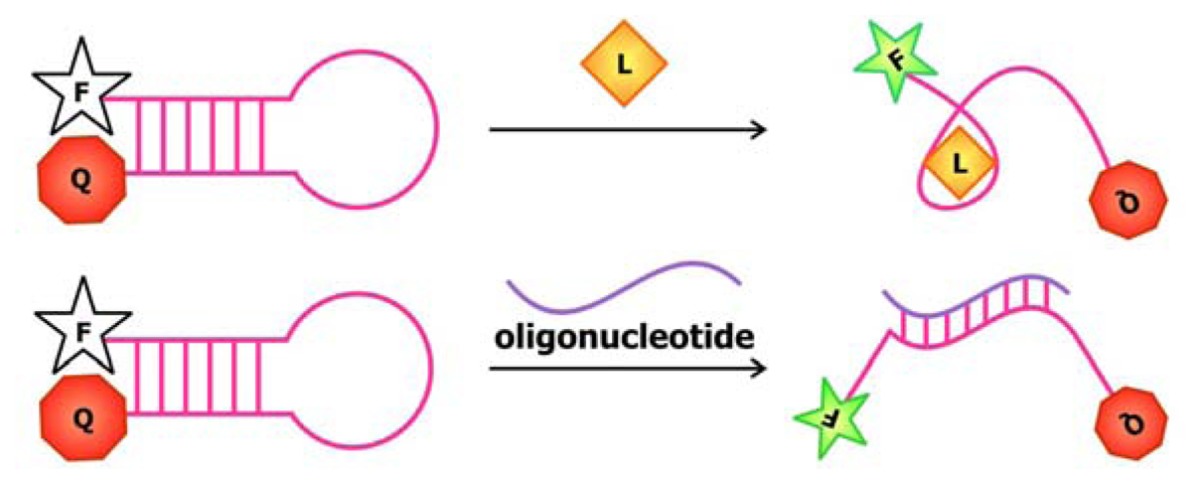 Often referred to as the molecular or aptamer beacon, this design involves the covalent linkage of a fluorophore at one end of the aptamer and a quencher at the other end [34]. In the absence of its target ligand, whether it is a small molecule (top panel) or a complementary oligonucleotide sequence (bottom panel), the aptamer is in its closed, hairpin conformation, thereby placing the quencher in close proximity with the fluorophore. Upon ligand binding, base-pairing in the hairpin is interrupted and the aptamer converts to its open conformation, thus increasing the distance between the fluorophore and quencher. The alleviation of fluorescence quenching leads to an intensification of the fluorescence signal.	Mg^2+^, Ca^2+^, K^+^ sensors [35]; PDGF-β sensor [36]; Tat protein (from HIV-1) sensor [37]
**C. Signaling by structure-switching** 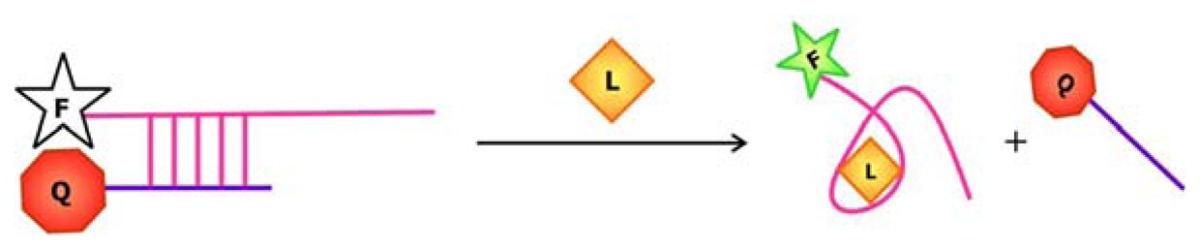 Similar to the aptamer beacon approach, the signal transducer domain of this signaling aptamer is also comprised of a fluorophore-quencher pair. Exploiting the ability of nucleic acids to form duplex structures with their complementary sequences, this signaling system requires the presence of a fluorophore-modified sequence encoding the aptamer against a target of interest and a quencher-modified complementary (antisense) sequence. Thus, in the absence of the target, base-pairing between the complementary sequences is favored, leading to a quenching of the fluorescence signal. In the presence of the target, the ligand-aptamer complex conformation is favored over the duplex conformation. This duplex-to-complex structure switching triggers the dissociation of the antisense sequence followed by an increase in the fluorescence signal.	ATP and thrombin structure-switching sensors [38]
**D. Signaling by fluorogenic reaction** 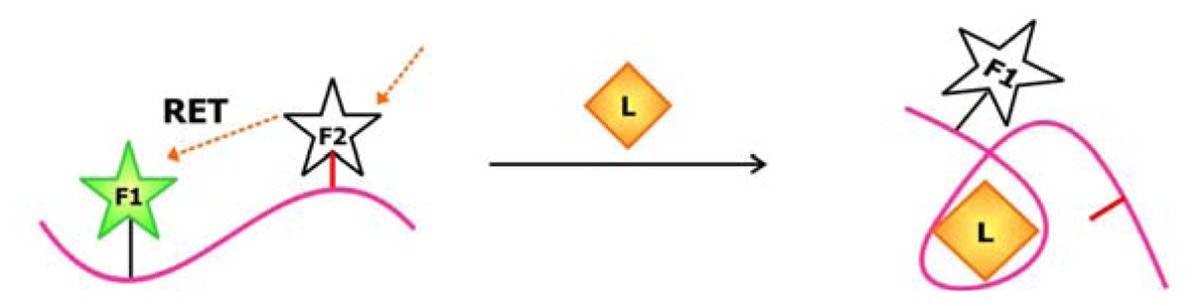 Using an aptamer sequence that has been modified with a fluorophore (F1) and a reactive group (denoted by the red line), this design favors the attachment of the second fluorophore (F2) to the reactive group in the absence of target binding. Under these circumstances, excitation of F2 would lead to a resonance energy transfer (RET) to F1, thus exciting F1 and producing a signal at its corresponding emission wavelength. However, this fluorogenic reaction is reduced in the presence of the target since target binding induces a conformational change to a structural arrangement that is unfavorable to F2 attachment.	ATP sensor [39]
